# Differential effects of phytotherapic preparations in the hSOD1 *Drosophila melanogaster* model of ALS

**DOI:** 10.1038/srep41059

**Published:** 2017-01-19

**Authors:** Francescaelena De Rose, Roberto Marotta, Giuseppe Talani, Tiziano Catelani, Paolo Solari, Simone Poddighe, Giuseppe Borghero, Francesco Marrosu, Enrico Sanna, Sanjay Kasture, Elio Acquas, Anna Liscia

**Affiliations:** 1 University of Cagliari, Department of Biomedical Sciences, Monserrato, 09042, Italy; 2 Istituto Italiano di Tecnologia (IIT), Department of Nanochemistry, Genova, 16163, Italy; 3 National Research Council (CNR), Institute of Neuroscience, Monserrato, 09042, Italy; 4 University of Cagliari, Department of Public Health, Clinical and Molecular Medicine, Monserrato, 09042, Italy; 5 University of Cagliari, Department of Life and Environmental Sciences, Cagliari, 09124, Italy; 6 Pinnacle Biomedical Research Institute, Bhopal, 462003, India

## Abstract

The present study was aimed at characterizing the effects of *Withania somnifera (Wse*) and *Mucuna pruriens (Mpe*) on a *Drosophila melanogaster* model for Amyotrophic Lateral Sclerosis (ALS). In particular, the effects of *Wse* and *Mpe* were assessed following feeding the flies selectively overexpressing the wild human copper, zinc-superoxide dismutase (hSOD1-gain-of-function) in *Drosophila* motoneurons. Although ALS-hSOD1 mutants showed no impairment in life span, with respect to GAL4 controls, the results revealed impairment of climbing behaviour, muscle electrophysiological parameters (latency and amplitude of ePSPs) as well as thoracic ganglia mitochondrial functions. Interestingly, *Wse* treatment significantly increased lifespan of hSDO1 while *Mpe* had not effect. Conversely, both *Wse* and *Mpe* significantly rescued climbing impairment, and also latency and amplitude of ePSPs as well as failure responses to high frequency DLM stimulation. Finally, mitochondrial alterations were any more present in *Wse*- but not in *Mpe*-treated hSOD1 mutants. Hence, given the role of inflammation in the development of ALS, the high translational impact of the model, the known anti-inflammatory properties of these extracts, and the viability of their clinical use, these results suggest that the application of *Wse* and *Mpe* might represent a valuable pharmacological strategy to counteract the progression of ALS and related symptoms.

Amyotrophic lateral sclerosis (ALS) is a neurodegenerative disorder characterized by the progressive loss of brain and spinal cord motoneurons (MNs). This affects the voluntary movements and relentlessly leads to paralysis of the whole body and finally death within 3 to 5 years of onset. Approximately 10% of ALS cases are inherited and around 20% of these familial cases are linked to mutations in the gene encoding the enzyme Cu/Zn superoxide dismutase 1 (SOD1)^1^,^2^. SOD1 is ubiquitously expressed in all cells and its best characterized function is the dismutation of the highly toxic superoxide anion radical into molecular oxygen and hydrogen peroxide, thus providing a defence against reactive oxygen species toxicity. To date about 150 ALS associated mutations in SOD1 gene have been identified, the majority of which are missense point mutations. Mutated SOD1 gene can acquire both gain and loss of function. In mice, lack of SOD 1 function does not lead to development of neurodegeneration, while its over-expression leads to ALS symptoms^3^,^4^,^5^. This evidence suggest that the toxicity related to these mutations depends on a toxic gain of function (GOF) rather than on a loss of function (LOF). The cellular and molecular mechanism through which these mutations (mutant SOD1) induce onset and progressive spreading of ALS pathology is complex and still not fully understood. This mechanism includes oxidative stress, mitochondrial damage, axonal transport impairment, glutamatergic excito-toxicity, activation of endoplasmic reticulum stress, RNA metabolism impairment, interaction with glial cells^6^ and protein aggregation^7^. In fact, SOD1 mutant proteins tend to be misfolded and form protease-resistant aggregates causing death of motoneurons^8^. Mutant SOD1 can seed misfolding and aggregation of endogenous wild-type SOD1 and transfer from cell to cell causing the intercellular transmission of disease through the central nervous system, a propagation mechanism similar to prion replication and spreading^9^,^10^.

It is worth noting that both insects and human beings share a number of genes and the organization and cellular function of the nervous system, making the *Drosophila melanogaster* a useful and powerful model for understanding the biological bases of human pathology and gaining evidence of high translational significance of such model and its potential therapeutic effectiveness. Accordingly, a number of neurodegenerative diseases, including Alzheimer’s^11^,^12^,^13^ and Parkinson’s diseases^14^,^15^,^16^,^17^,^18^,^19^ as well as ALS, object of this study^20^,^21^, are heuristically modeled in *Drosophila*. However, despite the significant progress in the knowledge of the biological bases of the degenerative diseases, the hope to find an efficient treatment has been, so far, elusive, as no treatment can halt their progression, with the partial exception of the antiglutamatergic Riluzole^®^.

In this context, given the results obtained in two different *Drosophila* models of Parkinson disease (PD-LRRK2-LOF and PD-PINK1^B9^-LOF) with the application of two methanolic extracts of parts of the medicinal plants, *Withania somnifera* and *Mucuna pruriens* widely used in the Ayurvedic medicine^17^,^18^,^19^,^22^,^23^,^24^ for their potential effects on treating many central nervous system disorders, we deemed to investigate their effects on ALS symptoms. In order to obtain experimental data, we proceed to set up a neurophysiological, biochemical and histological investigation using the hSOD1-GOF *Drosophila* model of ALS. Thus, based on the observations (1) that the PD-LRRK2 and ALS-SOD1 diseases share common oxidative stress- and neuroinflammation-based mechanisms and (2) that both *Withania somnifera* and *Mucuna pruriens* are endowed with potent anti-oxidative and anti-inflammatory properties, the results of the present study are expected to provide new data on the pathophysiology of ALS models and to suggest possible new vistas in the inflammatory, oxidative stress, genetic roles correlated to this neurodegeneration. In particular, given that neurodegenerative diseases are often associated with abnormal protein accumulation^25^,^26^, the mechanism by which ALS may take place is by the overexpression of human SOD1 in the motoneurons in agreement with Stathopulos *et al*.^27^ that could be regarded as a destabilizing solution condition resulting as a consequence in loss of mitochondrial functionality and also in altered vescicle trafficking. Thus, in particular, *Mpe* treatment might enhance bruchpilot synthesis similarly to what we found in another neurodegenerative disease in PD-PINK1^B9^ *Drosophila* mutants^18^, while *Wse* treatment might act by enhancing the vesicle trafficking as previously suggested in LRRK2^19^. Moreover, in the context of current knowledge about the mechanism of action of these drugs, even if they do not specifically regard ALS, a few references provide insight on the mechanism by which these compounds exert their anti-inflammatory effects^28^,^29^.

## Results

### *Wse*, but not *Mpe*, treatment enhances survival rate of hSOD1 flies

**Figure 1:**
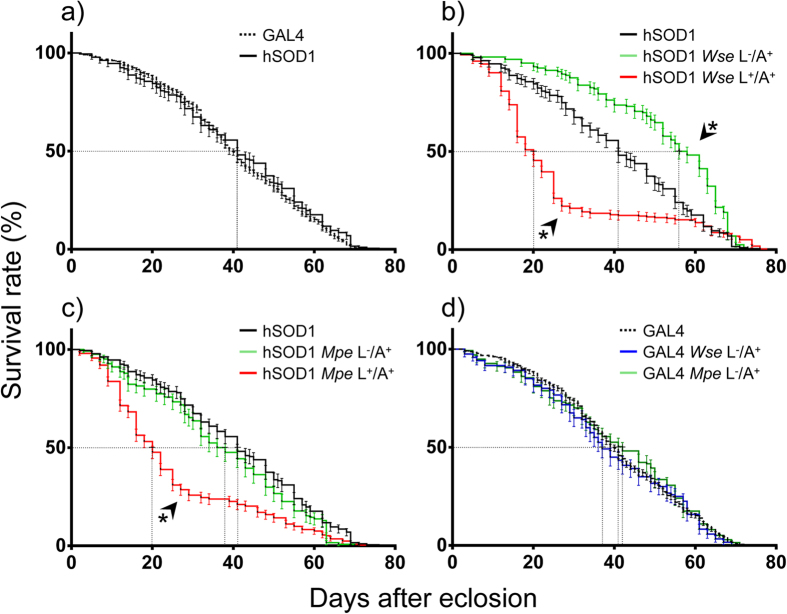
Effects of *Withania somnifera* extract (*Wse*) and *Mucuna pruriens* extract (*Mpe*) treatment on lifespan. (**a**) Lifespan, expressed as survival rate (%), of untreated isogenic flies (Gal4) and untreated mutant flies (hSOD1). (**b**) Lifespan of untreated hSOD1 compared to *Wse*-treated hSOD1, only when adults (L^−^/A^+^) and from their larval stage to the end of their life cycle (L^+^/A^+^). (**c**) Lifespan of untreated hSOD1 compared to *Mpe*-treated hSOD1, only when adults (L^−^/A^+^) and from their larval stage to the end of their life cycle (L^+^/A^+^). (**d**) Lifespan of untreated GAL4 control flies compared with *Wse*- and *Mpe*- treated flies only when adults (L^−^/A^+^). *Indicates p < 0.05 at Kaplan-Meier survival curves (Gehan-Breslow-Wilcoxon-Graph Pad Prism 5.01), (**b**) untreated hSOD1 compared to (L^−^/A^+^) and (L^+^/A^+^) *Wse*-treated hSOD1 and (**c**) untreated hSOD1 compared to (L^+^/A^+^) *Mpe*-treated hSOD1.

Figure 1a shows that the mutant hSOD1 that over expressed human SOD1 in the motoneurons exhibited no significant change in lifespan compared to GAL4. Accordingly to previous results, obtained with fly models of Parkinson’s disease^18^,^19^, the hSOD1 mutants were treated with *Wse* or *Mpe* at 0.1% w/w in their standard diet starting from larvae stage (L^+^/A^+^) or as adults only (L^−^/A^+^). As shown by Kaplan-Meier survival curves, administration of *Wse* as L^−^/A^+^ exerts great significant effects in mutant flies (Fig. 1b) with a significant increase in lifespan compared to not treated mutants. Surprisingly, the average lifespan of *Wse*-treated hSOD1 mutants was also longer than that of GAL4 (being 50% and 58% of survival at 40-days-old in GAL4 and in *Wse*-treated hSOD1 mutants, respectively). In contrast, *Mpe* administered as L^−^/A^+^ had no effect on the lifespan of mutant hSOD1 (Fig. 1c) (p > 0.05 at Gehan-Breslow-Wilcoxon test). As expected, neither *Wse* nor *Mpe* affected the lifespan of the GAL4 control flies (Fig. 1d). In addition, when administered as L^+^/A^+^, a dramatic decrease of the lifespan was induced by both *Wse* or *Mpe* (Fig. 1b,c).

### *Wse* and *Mpe* treatment ameliorates the climbing behaviour of hSOD1 flies

**Figure 2:**
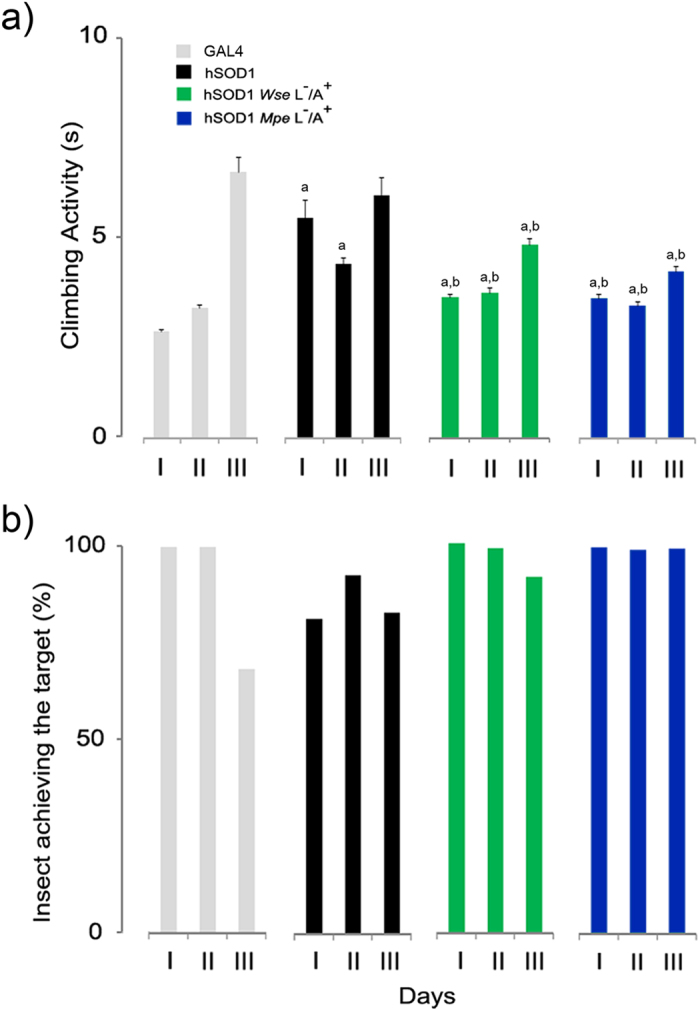
Effects of *Wse* and *Mpe* on climbing activity. **(a**) Climbing activity of adult GAL4 flies, untreated mutants (hSOD1) and *Wse*- and *Mpe-* treated hSOD1. The treatment was administered during the adult stage of mutants (L^−^/A^+^) and its effect was assayed at three different age steps (I: 3–6; II: 10–15; III: 20–25 days) of flies’ life-span. Values are average ± SEM. ^a^Indicates significant difference from Gal4 (Two-way ANOVA with HSD post-hoc test, p < 0.05); ^b^indicates significant difference from hSOD1 (Two-way ANOVA with HSD post-hoc test, p < 0.05). (**b**) Percentages of adult Gal4, hSOD1 and *Wse* L^−^/A^+^- and *Mpe* L^−^/A^+^- treated hSOD1 that could climb unto, or above, the line drawn at 6 cm from the bottom of the tube within 10 seconds.

As shown in Fig. 2a, hSOD1 mutants showed a significant increase in the climbing time in groups I and II compared to the GAL4 line, with a worsening trend with aging displayed at age stage III in which also GAL4 showed a significant slowing-down of climbing activity. Notably, flies overexpressing hSOD1 in motoneurons showed a significant impairment of climbing activity within the first week (age group I shown in Fig. 2) when compared with flies expressing dSOD1 i.e. GAL4. In order to further characterize the effects of the extracts on this genetic model of ALS, we tested climbing activity of flies administered with *Wse* or *Mpe* (Fig. 2a,b) (as L^−^/A^+^). Administration of *Wse* or *Mpe* was done in these experiments only in the condition L^−^/A^+^, suggested by the lifespan experiments which demonstrated that the administration to flies as L^+^/A^+^ resulted in a dramatic decrease of lifespan. Treatment of groups I-III of hSOD1 mutants with both *Wse* or *Mpe* (0.1% w/w) induced a recovery of motor disability compared to untreated mutants. Moreover, the percentage of flies that achieved the target (10 s) was very close to 100% in both *Wse*- and *Mpe*-treated flies, higher than those of untreated mutants and, surprisingly, also in respect to age-group III GAL4 flies (Fig. 2a,b).

### *Wse* and *Mpe* affect kinetic properties of evoked PSPs recorded from DLM in hSOD1 mutant flies

**Figure 3:**
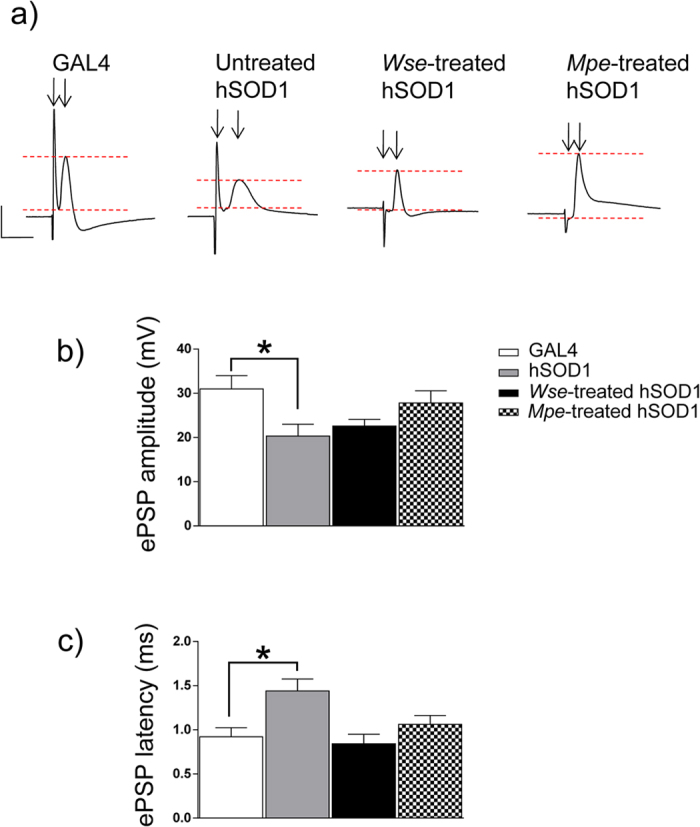
Effect of hSOD1 gene mutation and treatment with *Wse* or *Mpe* on ePSP latency and amplitude recorded from Drosophila DLM. **(a**) Representative traces obtained from four different flies of the different experimental groups in which ePSP latency is calculated as the time (ms) from stimulus application to the peak of PSP (black arrows) and PSP peak is calculated by measuring the maximal amplitude of the response starting from the baseline (red scatter lines). Scale bar, 20 mV/5 ms. (**b,c**) Bar graphs represent the mean ± SEM of PSP amplitude (mV) (**b**) and latency (ms) (**c**) recorded from flies treated with *Wse* and with *Mpe,* *P < 0.05 compared to GAL4, one-way ANOVA, followed by Bonferroni post-hoc test. N = 24 (GAL4), 16 (untreated-hSOD1), 21 (*Wse*-treated hSOD1) and 16 (*Mpe*-treated hSOD1).

In these experiments we first evaluated the potential changes in the function of the DLM neuromuscular junction of hSOD1 (group II) compared with GAL4 control flies. Basal kinetic properties (amplitude and latency) of evoked PSPs (ePSPs) recorded from the DLM after Giant Fibre System (GFS) electrical stimulation were analyzed. ePSPs recorded from DLM muscle of GAL4 animals had an average amplitude of 30.98 ± 3 mV and a latency of 0.92 ± 0.1 ms (Fig. 3a–c). Responses measured in hSOD1 mutants were characterized by a significant decrease in amplitude [20.32 ± 2.7 mV, one-way ANOVA, F (3,61) = 3.71, P < 0.05 vs GAL4 Bonferroni’s post-hoc] and a significant increase in latency [1.4 ± 1.1 ms, F(3,61) = 5.57, P < 0.01 vs GAL4 Bonferroni’s post-hoc] (Fig. 3a–c), suggesting that this mutation hampers the function of GFS-DLM muscle conduction. Interestingly, treatment of hSOD1 flies with *Wse* or *Mpe* reverted the reduction in ePSP amplitude as well as the increase in latency with values that were not significantly different to those observed in GAL4 flies (P > 0.05, one-way ANOVA, Bonferroni’s post-hoc) (Fig. 3a,c).

### *Wse* and *Mpe* ameliorate the ePSP responses to increasing stimulation frequency of GFS in hSOD1 mutant flies

**Figure 4:**
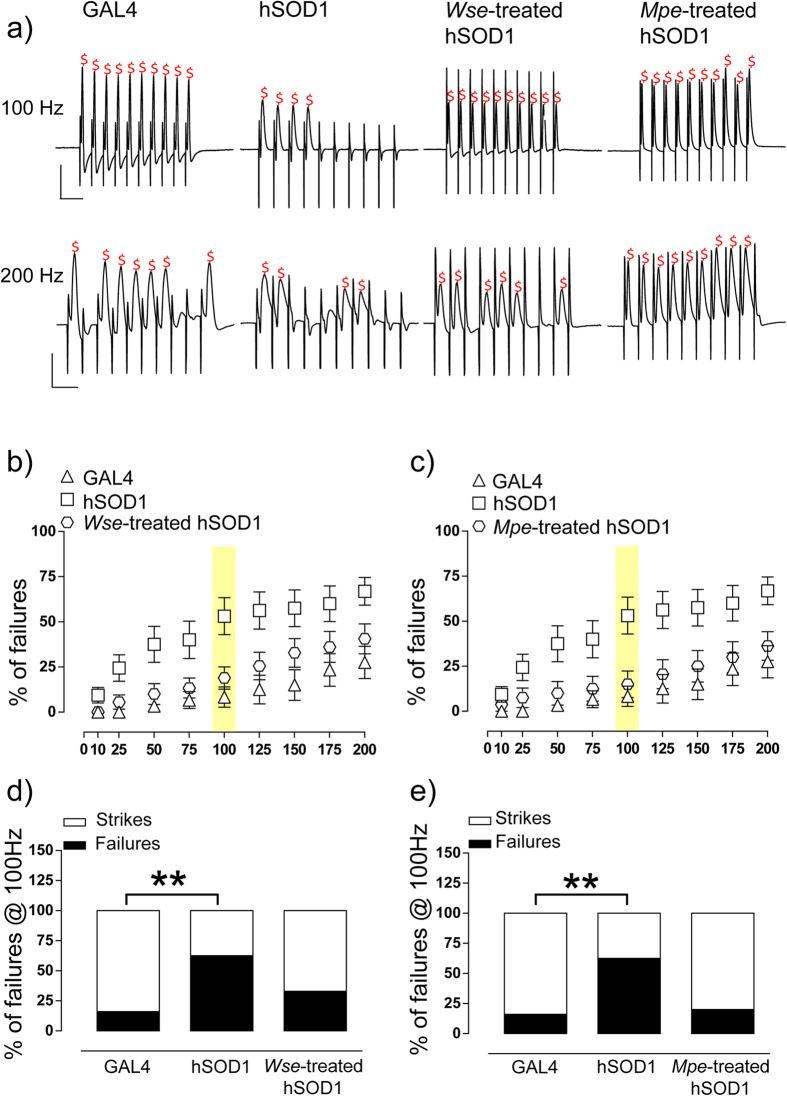
Effect of SOD1 gene mutation and treatment with *Wse* or *Mpe* on PSP response to “frequency of following” recorded in Drosophila DLM. (**a**) Representative traces obtained from four different flies of different experimental group in which PSPs were evoked in response to 10 stimulations at increasing train frequency of 10 consecutive stimuli. ^$^Indicates the detected response at 100 (top) or 200 Hz (bottom). Scale bar 20 mV/20 ms for 100 Hz and 20 mV/10 ms for 200 Hz. (**b,d**) Scatter plot graphs showing the changes in PSP amplitude following stimulation at increasing frequency (the effect at 100 Hz is highlighted in yellow). All values are expressed as the mean ± SEM of the % of failure observed in every train. In d the averaged % of failure was plotted of GAL4, hSOD1 and hSOD1 treated with *Wse*. **P < 0.01 compared to GAL4, unpaired t-test. (**c,e**) Scatter plot graphs showing the changes in PSP amplitude following stimulation at increasing frequency (the effect at 100 Hz is highlighted in yellow). All values are expressed as the mean ± SEM of the % of failure observed in every train. In e the averaged % of failure was plotted of GAL4, hSOD1 and hSOD1 treated with *Mpe*. **P < 0.01 compared to GAL4, unpaired t-test.

We further tested flies, from the different experimental groups, by recording the “frequency of following” which consisted in applying a train of 10 stimuli at different frequencies (from 10 to 200 Hz, with steps of 25 Hz) to GFS. As previously reported^19^,^21^,^30^, in control flies the number of failures increased depending on the increase in simulation frequency. In particular, in GAL4 flies a train of 10 stimulations delivered at 100 Hz induced a limited rate of failures (8.3 ± 5.6%), while a train at 200 Hz resulted in a predictive and consistent increase in failures percentage (27.5 ± 8.9*%, P < 0.05, one-way ANOVA, Bonferroni’s post-hoc) (Fig. 4a,b,d). Interestingly, when hSOD1 flies were exposed to the same protocol, the rate of failures at both 100 and 200 Hz resulted significantly enhanced with respect to GAL4 flies [F(2,41) = 6.8, P < 0.05, two-way ANOVA Bonferroni’s post-hoc] (Fig. 4a,b,d). Our findings are also consistent with behavioural data where hSOD1 mutant flies showed uncoordinated movements impairments that, in turn, could be related to the low probability of muscle contraction. As observed for the basal properties of ePSP, treatment with either *Wse* or *Mpe* was able to antagonize the increased rate of failures in response to train stimulations and the percentage of failures observed at 100 Hz showed values not significantly different from those observed in GAL4 flies (Fig. 4a–e). Surprisingly, both *Wse* and *Mpe* treatment were able to prevent the effect of the mutation making the response latencies and amplitude recorded in hSOD1-treated flies indistinguishable from those in the GAL4 flies.

**Figure 5:**
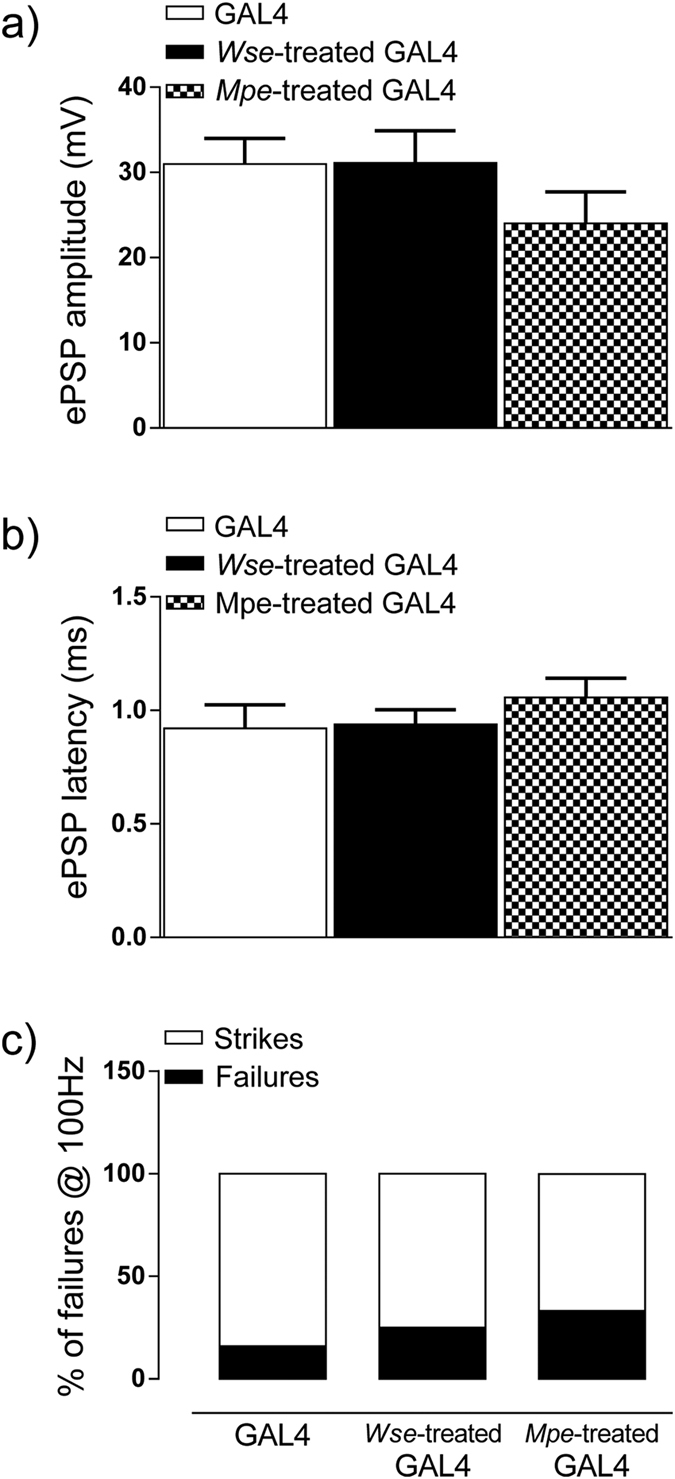
Effects of *Withania somnifera* extract (*Wse*) and *Mucuna pruriens* extract (*Mpe*) treatment on GAL4 on ePSP latency, amplitude and response to “frequency of following” recorded in *Drosophila* DLM. (**a,b**) Bar graphs, representing the mean ± SEM of PSP amplitude (mV) (**a**) and latency (ms) (**b**) recorded from GAL4 flies and GAL4 treated with *Wse* or *Mpe,* indicate that treatment failed to induce change of ePSP parameter when compared with untreated GAL4 (N = 24 (GAL4), 8 (*Wse*-treated GAL4), 7 (*Mpe*-treated GAL4); (**c**) Bar graph representing the averaged % of failure plotted in GAL4 and GAL4 treated with *Mpe* or *Wse,* indicate that the treatment fails to alter this parameter when compared with untreated GAL4.

Interestingly treatment with *Wse* and *Mpe* in GAL4 animals failed to change both sPSP amplitude and latency when compared with untreated GAL4 animals (one-way ANOVA, F (2,24) = 1.19, P > 0.05 vs GAL4 Bonferroni’s post-hoc) (Fig. 5a,b). Moreover, even for “frequency of following” responses, treatment with *Wse* and *Mpe* in GAL4 animals failed to change this response of sPSP when compared with untreated GAL4 animals (Fig. 5c).

### *Wse*, but not *Mpe*, treatment rescues the impairment of mitochondrial morphology in motoneurons of hSOD1 mutant flies

**Figure 6:**
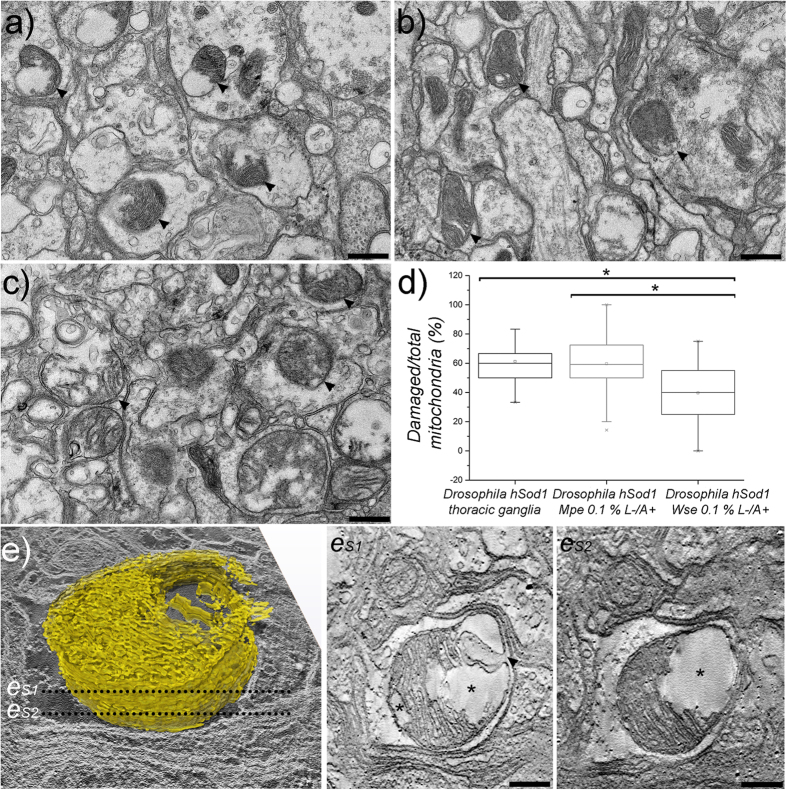
Electron microscopy. Representative TEM images of thoracic ganglia (T1–T2 regions) in *Drosophila* hSOD1 untreated (**a**) and treated with *Wse* L^−^/A^+^ (**b**) and *Mpe* L^−^/A^+^ (**c**) respectively. (**d**) Percentage of damaged versus total mitochondria in thoracic ganglia (T1–T2 regions) of *Drosophila* hSOD1 untreated (hSOD1, n mitochondria = 464) and treated with a 0.1% solution of *Mucuna* (hSOD1 *Mpe*, n mitochondria = 524) and *Whitania* (hSOD1 *Wse*, n mitochondria = 271) respectively. *t-Student test p-value < 0.01. (**e**) 3D model representing the reconstruction of a single mitochondrion in *Drosophila* hSOD1 thoracic ganglia (T1–T2 regions). The 3D model is set on a tomographic section. The es1 and es2 images are digitally inverted single tomographic slices corresponding to sections es 1 and es 2 in the 3D reconstruction. Arrowheads point to mitochondria. Asterisks point to damaged areas in the mitochondrion (see main text). Scale bars are 0.4 μm in (**a**,**b**) and (**c**), and 0.2 μm in es1 and es2.

Transmission (TEM) and scanning transmission electron microscopy (STEM) analysis were performed in untreated hSOD1 flies and hSOD1 mutants treated with 0.1% w/w *Wse* or *Mpe* as L^−^/A^+^. All flies analyzed were belonging to group II (10–15 days old) at the moment of morphological assessment. A high number of clearly damaged mitochondria with fragmented cristae, abnormally enlarged membranes and inhomogeneous electron transparent matrix have been observed by TEM and EM tomography in the T1- T2 regions of the thoracic ganglia in *Drosophila* hSOD1 mutants^21^ (Fig. 6a–e and Supplementary Video S1). Intriguingly, a similar density of damaged mitochondria has been observed in those mutants also after treatment with *Mpe* (Fig. 6c,d). On the contrary *Drosophila* hSOD1 mutants treated with *Wse* showed a significant reduction in the number of damaged mitochondria (Fig. 6b,d) compared with untreated hSOD1 flies. The treatment of the hSOD1 mutants with *Wse* was thus able to significantly decrease the number of damaged mitochondria in T1–T2 regions of thoracic ganglia rescuing also climbing as well as muscle electrophysiological parameters.

## Discussion

In agreement with Watson *et al*.^21^ the results of the present study disclosed that the survival curves of flies overexpressing hSOD1-GOF in motoneurons were not significantly impaired with respect to GAL4 control flies. As discussed in the review by Casci and Pandey^31^ the overexpression of SOD1 in different tissues may also be responsible of an improvement of life span thus, both the loss of expression of SOD1 and its overexpression may be responsible in affecting the lifespan. Interestingly, administration of *Wse* or *Mpe* to hSOD1 mutant flies resulted in differential effects also depending on the maturity stage of the flies. In particular, while both *Wse* and *Mpe* administration resulted in a reduced lifespan when administered to flies as L^+^/A^+^, only *Wse* increased the life duration of hSOD1 mutants when administered as L^−^/A^+^.

Impairment in lifespan following *Wse* administration to hSOD1 flies as L^+^/A^+^ agrees with our previous data regarding the *Wse* exposure to LRRK2 PD mutants leading us to the conclusions that *Wse* may have, after long term exposure or exposure at high concentrations, toxic effects^19^. Furthermore, the present observation after *Wse* administration to hSOD1 mutants as L^−^/A^+^ appears in agreement with the ability to prolong lifespan of LRRK2 PD mutants upon treatment as L^−^/A^+ ^19. This suggests that the widely accepted anti-inflammatory properties of *Withania somnifera*^32^ may represent the common mechanism underlying lifespan increase of hSOD1 (present data, Fig. 1) (TS50 (untreated hSOD1) = ~20 days versus TS50 (*Wse*-treated hSOD1) = ~60 days) and that of LRRK2 mutants^19^.

Notably, *Mpe* administration to hSOD1 mutants produced opposite results with respect to our previous study with *Mpe* administration to flies of another genetic model, the PINK1^B9^ PD mutants^18^. In fact, administration of *Mpe* to PINK1^B9^ PD mutant flies as L^+^/A^+^ resulted in a significant rescue of the lifespan, whereas its administration to L^+^/A^+^ hSOD1 resulted in a dramatic lifespan reduction. On the other hand, when *Mpe* was administered as L^−^/A^+^ the extract failed to affect this parameter in both mutants. Anyway, the overall results, suggest that the genetic and metabolic pathways of these two mutants differ significantly as do the effects of these phytotherapic extracts, that, we recall, do not affect the lifespan of GAL4 control flies when administered as L^−^/A^+^.

Also, the observation that both extracts protect against the development of motor impairment suggests that *Wse* and *Mpe* can have a positive impact on complex climbing behaviour, although involvement of distinct pathways either in terms of neuronal circuitry and metabolic machinery is still unclear. This seems in agreement also with our previous studies with LRRK2^19^ and PINK1^B9 ^18 genetic models of PD in which we found that *Wse* and *Mpe*, similarly to the present data at stage II of age, significantly ameliorated climbing behaviour.

Interestingly, both *Wse* and *Mpe* administered to flies as L^−^/A^+^ were able to protect from the climbing impairment in an age-independent manner and, surprisingly, also protected group III hSOD1 mutant flies from the age-related spontaneous impairment as suggested by the observation that we did not detect any significant difference between the dSOD1 (i.e. GAL4) and untreated hSOD1 flies. The observation that in our conditions the motor impairment appears as early as within the first five days (group I) and remains almost constant (Fig. 2), is at variance with what Watson *et al*.^21^ found in flies expressing WT (not overexpressed) hSOD1 which showed a progressive loss of climbing starting at 21 days as compared with dSOD1 controls. In this respect, the difference we found is likely imputable to differences in the expression level of hSOD1. Accordingly, Watson *et al*.^21^ reported in *Drosophila* an accumulation of hSOD1 in motoneurons that increased with the age.

Electrophysiological data showed that hSOD1 over expression in motoneurons was associated with a significant increase in latency of ePSPs with a parallel and significant decrease in amplitude when compared to GAL4 animals, an effect no longer present in hSOD1 flies fed both *Wse* and *Mpe*. Conversely, neither *Wse* nor *Mpe* affected the GAL4 electrophysiological parameters.

These data are in agreement with previous reports in which hSOD1 flies showed an impairment of synaptic transmission that become progressively defective^21^. Moreover, differently from our data, Watson and colleagues^21^ reported such kind of impairments only in 55 days old flies in contrast to their 10 days old WT hSOD1 flies which showed a normal synaptic response. The differences between the present data and those of Watson *et al*.^21^ can be attributable to the differences in the genetic background of mutants belonging to these different studies. In fact, in this regard, it should be kept in mind that Watson and colleagues^21^ performed their data on WT hSOD1 or mutation of this gene while we recorded from mutant flies in which hSOD1 was overexpressed and this may lead to a more pronounced impairment in motor neurons, the only cells in which the mutation occur, that may appear earlier in life than in Watson’s flies.

The lower amplitude and the higher latency of ePSP shown by hSOD1 muscle responses may be related to a decrease in neuronal conduction and a possible related decrease in the probability of neurotransmitter release from presynaptic terminals. Our findings suggest that the effects of *Wse* and *Mpe* on the functional changes associated with the hSOD1 over expression seem clearly connected to beneficial aspects of these treatments. Furthermore, hSOD1 flies showed an increase in failure of PSPs after increasing frequency stimulation compared with GAL4 flies. Even partially different from other reports^21^, that showed such modification later in life, our data are strongly consistent with climbing attitude results that show an impairment that appear earlier in life. In addition, we found that both *Wse* and *Mpe* were effective also in reverting the changes on train frequency responsiveness observed in hSOD1 flies. Interestingly, the effect of *Wse* on genetically-mediated impairments on percentage of failures at increasing frequency stimulation is in agreement with our previous data on LRRK2 mutants^19^. Furthermore, the results on the percentage of failures following *Mpe* treatment are in agreement with the morphological observations of our previous study made with PINK1^B9^ PD mutants^18^ in which we observed an increased expression of T bars. These observations point to the ability of these extracts to overall impact, perhaps through different mechanisms, on impaired release of neurotransmitters from pre-synaptic terminals.

Locomotor impairment as well as altered muscle contraction here presented well correlate with the presence of a high density of damaged mitochondria in the hSOD1 T1-T2 regions of thoracic ganglia. Boillée *et al*.^2^ by means of *in vivo* studies, emphasizes the importance of the glia as well as the glia-neurons interaction in the development of ALS. Although on the basis of our results we cannot exclude an involvement of glia or specific glia-neuron interactions in ALS, our EM analysis showed that mainly thoracic ganglia neurons, characterized by the presence of T-Bars in their presynaptic terminals, showed damaged mitochondria. The damaged mitochondria at level of presynaptic terminals may be in agreement with electrophysiological data showing that hSOD1 overexpression is also related to an impaired postsynaptic response to high frequency stimulation of presynaptic terminals that fired to DLM motoneurons.

In agreement with other studies^33^,^34^,^35^,^36^,^37^,^38^ our results point to a possible link between SOD1, mitochondrial dysfunction and ALS. Intriguingly, we showed that the over-expression of the wild type hSOD1 also causes mitochondrial alterations. Thus, not only mutation in hSOD1 but also alteration in its expression may lead to mitochondrial dysfunction and possibly drive to ALS symptoms. Interestingly it has been shown that overexpressed mutant SOD1 mislocalize with mitochondria affecting their functions and contributing to the degeneration of motoneurons leading to ALS^38^,^39^. Although the proposed model is appealing, further experiments are needed to clarify the mechanisms by which wild type hSOD1 overexpression affects mitochondria functioning. We hypothesize that an overexpression of human SOD1 in the motoneurons could be regarded as a destabilizing solution condition^27^ and mitochondria are altered as a consequence.

Treating the hSOD1 mutants with *Wse* was able to significantly decrease the number of damaged mitochondria in T1-T2 thoracic ganglia region rescuing also climbing as well as muscle electrophysiological parameters. Intriguingly, *Wse* exhibited a similar rescue effect on damaged mitochondria in LRRK2 loss-of-function *Drosophila* model of PD^19^. The use of this drug as a therapy is really promising for many pathologies, but no data are available specifically on *Withania somnifera* extract against ALS, even if a number of papers are present regarding the effects of active molecules present in the extract according to the widely accepted anti-oxidant activity (glycowithanolides sitoindosides VII-X and withaferin A)^32^,^40^,^41^,^42^. More recently, Patel *et al*.^43^ reported that Withaferin A reduces the levels of misfolded SOD1 in a mouse model of ALS. Instead, the present work specifically used *Withania somnifera* and *Mucuna pruriens* extracts against ALS-SOD1. Moreover, the present work indicates that *Drosophila melanogaster* is a powerful model of ALS, convenient because of the reproducibility, the low cost and a relatively short life-span with respect to other models. More importantly, it indicates that this organism can be used as a model in studying phytoterapic approaches to ALS. Specifically *Withania somnifera* extract presents, compared to the single active molecules above reported, different advantageous being more safety, as it is used in Ayurvedic medicine since many centuries, cheaper as it does not need extraction costs and easy to be administered. As already cited, *Withania somnifera* powder is considered very safe for values ranging from 0.001 mg to 1000 mg per kg. of body weight, showing modest tranquilizers and hypotensive effects at 25 mg/kg^32^.

Additional pre-clinical studies are necessary in order to verify the efficacy of this drugs on ALS-TDP43 while the present data strongly support the use of this extract to counteract the ALS-symptoms in humans. In this regard, we acknowledge that clinical studies will be required in order to characterize the optimal conditions (dosage and duration) at which these drugs might be successfully used to counteract this terrible disease.

In this respect, the observation that the administration of *Wse* and *Mpe* to flies as L^+^/A^+^ induces a further reduction of lifespan as compared to WT controls and untreated hSOD1 indicates that these phytoterapics may exert their effects -as a drug- following a hormesis-like dose-response curve^44^, similarly to what observed in the LRRK^WD40^ *Drosophila* PD mutant^19^, and further highlights the need to assess the proper concentration and duration of treatment.

Unlike the Wse treatment, the *Mpe* one on *Drosophila* overexpressing hSOD1 did not rescue mitochondrial impairment in the thoracic ganglia. This result is surprising since the same treatment well rescued climbing as well as muscle electrophysiological parameters. Moreover, *Mpe* well restored mitochondrial impairment in the olfactory bulbs of PD-PINK^B9^ mutants^17^. These results, that need further experiments to be fully understood, suggest that *Wse* and *Mpe* rescue mitochondrial damage probably through different molecular mechanisms. This is suggested also by the significantly different quantitative composition of *Mpe* that includes, among others, a number of anti-oxidant agents such as Fe^2+^ and glutathione responsible of preventing the consequences of both hSOD1 over-expression as well as mitochondrial functional defects due to silencing of PINK1^18^. In addition several evidences suggest that a common path in different neurodegenerative desease is the increase in the formation of inflammatory markers such as pro-inflammatory cytokines including interleukin-6 and tumor necrosis factor-α, nitric oxide and reactive oxygen species (ROS) (see D’Ambrosi *et al*.^45^ for review) contributing to mitochondrial function and inducing cell death. In this regard, it has widely reported that *Wse* show a potent inhibitory effect on inflammatory markers such as ROS in a mouse model of lupus^46^,^47^. Interestingly *Wse* reduced rotenone-induced oxidative impairment and mitochondrial respiratory chain enzymes and such impairments were responsible for reduced locomotor deficits and lethality in a *Drosophila melanogaster* model of Parkinson induced by rotenone, a naturally occurring common pesticide which specifically inhibits mitochondrial complex-I activity^48^. We here speculate that *Wse* as well as *Mpe* may affect the activity of mitochondrial complex-I inhibiting the negative action induced by SOD1 mutation onto mitochondrial function.

Taken together, our results demonstrate that an overexpression of wild hSOD1, in *Drosophila melanogaster* flies, is able to alter the motor coordination of mutant animals, an effect that is already present in young animals and remains even in adulthood. The positive effect of *Wse* as well as *Mpe*, results in an improved performance (climbing) and in a recover of normal electrophysiological function of locomotor system. Moreover, on the basis of our results it seems clear that both Parkinson Disease (PD) and Amyotrophic Lateral Sclerosis share mitochondrial dysfunction even if they differ in both genetic and metabolic pathways. Finally, given the role of inflammation in the development of ALS, the known anti-inflammatory properties of these extracts and the viability of their clinical use, these results suggest that the application of *Wse* and, to lesser extent, of *Mpe* might represent a valuable pharmacological strategy to counteract the progression of ALS and related symptoms.

## Methods

### Flies

We used the GAL4-UAS binary system^49^,^50^ to over-express human SOD1 (hSOD1) specifically in motoneuron cells of *D. melanogaster*. For these experiments, we crossed GAL4D42 (dSOD1, #42737; from now on reported as GAL4) with UAS-hSOD1 (hSOD1, #33656, from Bloomington Stock Center; Fly Base: http://flybase.bio.indiana.edu) flies. After emergence from pupae, GAL4 (isogenic controls) and hSOD1 mutant male flies were reared on a standard cornmeal-yeast-agar medium in controlled environmental conditions (24–25 °C; 60% relative humidity; light/dark = 12/12 hours).

In detail, a part of GAL4 and hSOD1 mutants, four groups of mutant flies were reared on a standard medium supplemented with *Withania somnifera (Wse*) or *Mucuna pruriens (Mpe*) extracts (kindly provided by Natural Remedies Pvt. Ltd., Bangalore, India). In agreement with our previous studies^18^,^19^ hSOD1 mutants were supplied with *Wse* or *Mpe* at 0.1% w/w concentrations both as larvae and adults (L^+^/A^+^) or as adults only (L^−^/A^+^). Moreover, lifespan of GAL4 *Wse*- and *Mpe*-treated flies as L^−^/A^+^ was analysed.

The effects of *Wse* or *Mpe* were assayed at different age steps (I: 3–6; II: 10–15; III: 20–25 days old). The experiments on life span, using different time of administration at the same concentrations of *Wse* or *Mpe* (see below in Survival curves) provided the basis for selecting the time of drug administration at which conduct the behavioural, electrophysiological and morphological experiments. In particular, based on lifespan results, behavioural, electrophysiological and transmission electron microscopy (TEM) were restricted to group II flies after 0.1% w/w *Wse* or *Mpe* administration as L^−^/A^+^. Standard genetic procedures were used during the study.

### Survival curves

As in *Drosophila* mutants for Parkinson’s disease both *Wse* and *Mpe* showed effects dependent on the duration of the treatment; *Wse* or *Mpe* standardized methanolic extracts were administered in two different modalities: as adults (L^−^/A^+^) only or as larvae and adults (L^+^/A^+^) with the aim of selecting the optimal *Mpe* and *Wse* administration time. In detail, flies were grown on standard diet supplemented with 0.1% w/w concentrations of *Wse* or *Mpe*. Cohorts of 60 flies (10 flies/tube) from each experimental group (i.e. GAL4, *Wse*-untreated, *Wse*-treated hSOD1, *Mpe*-untreated and *Mpe*-treated hSOD1), were monitored every 2 days for their survival. Mortality was analyzed using Kaplan-Meier survival curves and the statistical comparisons were made with a Gehan-Breslow-Wilcoxon test. All experiments were done in triplicate. All experiments were done in triplicate, except for Wse- and Mpe-treated Gal4 flies, that were done in duplicate.

### Climbing assay

The climbing assay (negative geotaxis assay) was used to assess locomotor ability both in GAL4 control flies and in the hSOD1 mutant ones as already reported by Liu *et al*.^51^, Poddighe *et al*.^17^,^18^ and De Rose *et al*.^19^.

Climbing data were obtained from different age groups (I: 3–6; II: 10–15; III: 20–25 days old) of untreated-GAL4, *Wse*-untreated, *Wse*-treated, *Mpe*-treated and *Mpe*-untreated hSOD1 mutants. Cohorts of 60 flies from each experimental group were subjected to the assay. Flies were placed individually in a vertically-positioned plastic tube (length 10 cm; diameter 1.5 cm) and tapped to the bottom. Climbing time (seconds) was recorded upon crossing a line drawn at 6 cm from the bottom. The number of flies that could climb unto, or above, this line within 10 seconds was recorded and expressed as percentage of total flies. Data were expressed as average ± standard error of the mean (SEM) from three experiment replications. Statistically significant differences (p < 0.05) were analyzed between GAL4 vs. hSOD1 and between untreated-hSOD1 vs. treated ones by means of the one-way ANOVA followed by HSD post-hoc test.

### Electrophysiological recordings

At the time of experiments, flies from the different experimental groups at age step II (10–15 days) were anesthetized by using CO2 and tightly anchored to a wax support with ventral side down, as previously reported^30^, and visualized under a stereomicroscope. In order to activate the GFS of the fly, two tungsten stimulating electrodes, connected to a stimulator (Master 8, A.M.P.I, Jerusalem, IL, USA) triggered by a stimulus isolation unit (DS2A, Digitimer Ltd., Hertfordshire UK), were placed into both eyes of the fly. Stimulus intensity was increased until the postsynaptic potential response was observed and maximal stimulation intensity was not greater than 10 V. Moreover the range of stimulation was very wide (the positive artefact, that represent the stimulation intensity, is perceptible before every single) and no correlation between stimulation intensity and type of animal tested was observed (data not shown). A ground tungsten wire was placed into the fly abdomen. A borosilicate recording electrode, shaped by a horizontal puller (P97, Fleming Brown, Sutter Instruments, Novato, CA, USA) with a resistance of 4–5 MΩ when filled with 3 M KCl, was placed into the right or left backside of the fly along the 45a and 45b fibres of the Dorsal Longitudinal Muscle fibres (DLMs). Evoked post-synaptic potentials (PSPs) were recorded with an Axopatch 2-B amplifier (Axon Instruments, Foster City, CA), filtered at 0.5 kHz and digitized at 1 kHz. PSPs were recorded in bridge mode, measured using peak and event detection software pCLAMP 8.2 (Axon Instruments, Foster City, CA), and analyzed off-line by pCLAMP fit software (Axon Instruments, Foster City, CA). All recordings were obtained from at least 10 different flies belonging to each experimental group. Experiments were blind to the treatment. Electrophysiological experiments were performed by applying a protocol consisting in a single GFS stimulation, delivered every 20 s, followed by PSP recording. The “frequency of following” was determined by delivering trains of 10 stimuli at increasing frequencies (from 10 to 200 Hz) and the failures, as the percentage of lacking responses at each train, were calculated. As previously published^19^ we also evaluated the amplitude (peak of the PSP expressed in mV) as well as the latency (interval between the stimulating artifact and the time at PSP peak, expressed in ms) of the first PSP evoked by the 10 Hz stimulation train. Data are expressed as mean +S.E.M. and analyzed by one or two-way ANOVAs followed by Bonferroni’s post-hoc tests.

### Electron microscopy analysis

Untreated hSOD1, *Wse*- and *Mpe*-treated hSOD1 mutants were anesthetized using CO2 and carefully decapitated. Once rapidly removed, the thoracic ganglia were fixed in a mixture of 2% glutaraldehyde and 2% paraformaldehyde in 0.1 M cacodylate buffer, washed several times in the same buffer, post-fixed in 1% osmium tetroxide in distilled water for 2 hours, and stained overnight at 4 °C in an aqueous 0.5% uranyl acetate solution. After several washes in distilled water, the samples were dehydrated in a graded ethanol series, and embedded in SPURR resin. Semi-thin coronal sections of the whole ganglion were cut with a Leica EM UC6 ultramicrotome, stained with toluidine blue and observed with a Leica DM2700 P light microscope. Sections of about 70 nm corresponding to portions of the thoracic ganglia were cut with a diamond knife on a Leica EM UC6 ultramicrotome. Transmission electron microscopy (TEM) images were collected with a FEI Tecnai G2 F20 (FEI Company, The Netherlands) and a Jeol JEM 1011 (Jeol, Japan) electron microscopes and recorded with a 1 and 2 Mp charge-coupled device (CCD) camera (Gatan BM Ultrascan and Gatan Orius SC100, respectively). The number of damaged mitochondria within the thoracic ganglia T1 and T2 regions (expressed as percentage of the total number of mitochondria/sampled area) was evaluated in untreated hSOD1, *Wse*-and-*Mpe*-treated hSOD1 mutants. More than 2300 mitochondria were randomly sampled on 227 non-overlapping micrographs at a final magnification of 6000x, corresponding to a total sampled area of more than 2800 μm^2^. Damaged mitochondria were recognized for the presence of swollen external membrane, clearly fragmented cristae and inhomogeneous electron transparent mitochondrial matrix. The mean differences were tested using a two-tailed t-test and a p < 0.01 level was considered statistically significant. EM tomography was performed in scanning TEM (STEM) using a high angular annular dark field (HAADF) detector on 400 nm sections of *Drosophila* hSOD1 the thoracic ganglia T1–T2 region. The tilt series were acquired from ± 65 degree tilt range using a Saxton tilt scheme with 3-degree increment at 0 tilt. A final magnification of 28000x was used, corresponding to a pixel size of 3.6 nm. Computation of tomograms was done with the IMOD (version 4.8.40) software package^52^. Isosurface based segmentation and three-dimensional visualization on unbinned and unfiltered tomograms were performed using the Amira software package (FEI Visualization Science Group, Bordeaux, France).

## Additional Information

**How to cite this article**: De Rose, F. *et al*. Differential effects of phytotherapic preparations in the hSOD1 *Drosophila melanogaster* model of ALS. *Sci. Rep.* **7**, 41059; doi: 10.1038/srep41059 (2017).

**Publisher's note:** Springer Nature remains neutral with regard to jurisdictional claims in published maps and institutional affiliations.

## Supplementary Material

Supplementary Video S1

Supplementary Information
