# Supplementary material for: Differential effects of phytotherapic preparations in the hSOD1 Drosophila melanogaster model of ALS
Source: Sci Rep. 2017 Jan 19;7:41059. doi: 10.1038/srep41059 (PMC5244478; doi:10.1038/srep41059)
Supplement: Supplementary Information [file srep41059-s2.doc]

**Differential effects of phytotherapic preparations in the hSOD1 *Drosophila melanogaster* model**

**of ALS**

**Francescaelena De Rose, Roberto Marotta, Giuseppe Talani, Tiziano Catelani, Paolo Solari, Simone Poddighe, Giuseppe Borghero, Francesco Marrosu, Enrico Sanna, Sanjay Kasture, Elio Acquas, Anna Liscia**

**Supplementary Video S1.** Tomographic reconstruction and resultant 3D model of a damaged mitochondrion in *Drosophila* hSOD1 thoracic ganglia (T1-T2 regions).
